# Multiple Hidden Markov Model for Pathological Vessel Segmentation

**DOI:** 10.1155/2018/9868215

**Published:** 2018-12-10

**Authors:** Xin Hu, Deqiong Ding, Dianhui Chu

**Affiliations:** ^1^School of Computer Science and Technology, Harbin Institute of Technology at Weihai, Weihai 264209, China; ^2^School of Computer Science and Technology, Harbin Institute of Technology, Harbin 150001, China

## Abstract

One of the obstacles that prevent the accurate delineation of vessel boundaries is the presence of pathologies, which results in obscure boundaries and vessel-like structures. Targeting this limitation, we present a novel segmentation method based on multiple Hidden Markov Models. This method works with a vessel axis + cross-section model, which constrains the classifier around the vessel. The vessel axis constraint gives our method the potential to be both physiologically accurate and computationally effective. Focusing on pathological vessels, we reap the benefits of the redundant information embedded in multiple vessel-specific features and the good statistical properties coming with Hidden Markov Model, to cover the widest possible spectrum of complex situations. The performance of our method is evaluated on synthetic complex-structured datasets, where we achieve a 91% high overlap ratio. We also validate the proposed method on a real challenging case, segmentation of pathological abdominal arteries. The performance of our method is promising, since our method yields better results than two state-of-the-art methods on both synthetic datasets and real clinical datasets.

## 1. Introduction

Automatic vessel segmentation in three-dimensional (3-D) medical computed tomography (CT) images plays a fundamental role in many clinical fields: study of anatomical structure [1], quantification of vascular diseases (stenosis, occlusion, and calcification) for clinical diagnosis [2], surgery planning [3], and patient-specific flow simulations [1]. Vessel segmentation can help clinical workers to have an intuitive impression of vessels and blood supply. Based on the vessel segmentation, clinical workers can also establish the patients' response to treatment and determine the stage of diseases, to further plan a minimally invasive surgery. All these applications ask for a competent segmentation technique, which has the capability of segmenting vessels accurately, not only for normal vessels but also for vessels with the presence of pathologies.

In recent years, many methods [1–3] have been proposed to segment vessels with the presence of pathologies. These methods can be roughly classified into three categories: (a) feature based segmentation approaches [4, 5], which have been proven to be efficient in detecting vessels at different scales; (b) tracking based segmentation approaches [6–8], which have the capability to be robust against noise; and (c) model based approaches [9–12], which resolve subsequent two-dimensional (2-D) slices of vessels using tubular shape priors.

By targeting the curvilinear structures, the feature based segmentation approaches are efficient in identifying vessels from other tissues [5, 13–18]. They have several common detection procedures: firstly, assuming that vessels have identifiable curvilinear structures and then calculating the eigenvalues of the Hessian matrix to traverse the whole 3-D image at different scales, by convolving with 3-D Gaussian filters. The standard deviation of the Gaussian filter defines the detecting scale. Third, a response function based on these eigenvalues is constructed, which can detect the curvilinear structures locally at a certain scale. Since the curvilinear structures signed to vessels are significantly different from other tissues, the feature based segmentation approaches can recognize vessels from other nonvessel structures, such as planar structure, blob, noise, or no structure. The response consisting of eigenvalues can represent the local structure, when it comes to its maximum over different scales [5, 16].

However, the feature based segmentation approaches do not produce direct vessel segmentation, they output responses instead of segmentation. As a result, their results can be used in structure analysis, where many researchers consider it as the advantage of the feature based segmentation approaches [19, 20], especially in combining the tracking based segmentation approaches. Based on the responses of the feature based segmentation approaches, the tracking based segmentation approaches resolve subsequent two-dimensional (2-D) slices of vessels, by using the tubular shape priors [19, 20]. Based on the tubular shape structure, Tyrrell et al. [21] propose another 3-D structure, 3-D cylindroidal superellipsoids, combining with the local regional statistics method. This tracking methodology extracts topological information from microvasculature networks.

The tracking based segmentation approaches are shown to be robust against noise [19–21]. However, the shape priors they use are too exclusive [22], which lead to false detection with the complex vessel boundaries. Targeting at this challenge, the statistical mixture models have been proposed [22–24]. They combine the statistical mixture models with expectation-maximization algorithm, to detect complex vessel boundaries. However, these approaches ask for an accurate parametrical estimation or nonparametric modeling. As a result, they all rely on the second-order derivative information, which is associated with the principal curvatures of image intensities. They suffer from sensitivity caused by local deformations, which are considered as pathologies in this work.

In the past decade, after many sophisticated vascular segmentation algorithms have been proposed, there are still several challenges remaining unsolved, such as detecting vessel radius with the presence of pathologies (stenosis and occlusion), delineating vessel accurately with low contrast vessel boundary, and distinguishing vessels from vessel-like tissues. All these challenges are caused by pathologies, especially by the vessel calcification. As far as we know, the accurate delineation of pathological vessel is valuable in clinical fields. However, the proposed challenges may lead to false segmentation, which makes the vessel segmentation useless. For further information, several good reviews can be found in [1–3].

Addressing these unsolved challenges, we propose a Multiple Hidden Markov Model (MHMM) for pathological vessels, by taking the advantages of the redundant information embedded in multiple vessel-specific features and the good statistical properties coming with the Hidden Markov Model (HMM). As we target at pathological vessels, the shape complexity of obscure vessel boundary may not be captured by one single existing vessel-specific feature. Thus, we dig the redundant information embedded in multiple vessel features automatically by using multiple HMMs and try to combine multiple vessel features optimally by employing the good statistical properties of HMM. Different from most of the other researchers, we build the MHMM to describe the transition course from the inner vessel to the outside of vessel. By doing so, the MHMM has the capability to delineate the obscure vessel boundaries accurately.

This paper is organized as follows.  Section 2 introduces the proposed method MHMM.  Section 2.1 details the training samples and the testing samples we use in this work. The multiscale vessel-specific feature set is followed in Section 2.2. Then we present the details of training a single HMM in Section 2.3. The MHMM combining multiple vessel features with multiple HMMs is given in Section 2.4. The evaluation datasets, the evaluation methods, and the experimental results are shown in Section 3. In Section 4, we discuss the advantages and disadvantages of our method, possible improvements, and future work. Finally, we conclude the paper in Section 5.

## 2. Method

This paper describes a Multiple Hidden Markov Model (MHMM) for abdominal artery with the presence of pathologies, which takes two advantages: (1) the redundant information included in multiple vessel features and (2) the good statistical properties of Hidden Markov Model (HMM) [25]. First, we extract vessel axis from a gray-scale 3-D CT scan, by using our previous method [9]. Then, along this extracted vessel axis, voxel series are generated on the cross-sections for training and testing purposes. Subsequently, multiple vessel features are calculated for each voxel in the voxel series. Finally, a MHMM is trained based on these vessel features. The trained MHMM can detect vessel boundary with the presence of pathologies, by expressing the similarity marching degree (posterior probability).

### 2.1. Training Samples and Testing Samples

We randomly selected 50 CT datasets of abdominal artery from 120 CT datasets, which were collected by our collaborators (the clinical workers from the 2^*nd*^ affiliated Hospital of Harbin Medical University). These 120 CT datasets were collected from 120 patients, 73 males and 47 females with mean age 65 years (min 58, max 91), who entered our study nonconsecutively from October 2014 to October 2017. These 50 selected CT datasets are known with aortoiliac stenosis, calcification, and occlusion.

Two expert raters were asked to segment the datasets. We understand that the ground truth segmentations of those clinical datasets do not exist, even segmented by multiple experts. Thus, we developed a graphical user interface (GUI) in our lab [9] to help the experts mark different tissues in 3-D CT images, including pathologies. With this GUI, the user can do the marking job in two stages: propagating stage and checking stage. In the propagating stage, the GUI can propagate to mark the target tissue in 3-D CT image automatically, with seed points given by experts. In the checking stage, the GUI helps the user to cine-page through the CT slices, scroll in and out of individual tissue, adjust window setting, and zoom to improve visualization. As a result, the user can check the propagating result and set new seed points. By using this GUI, two expert raters were asked to mark vessels and pathologies (calcification and iliac stent).

**Figure 1:**
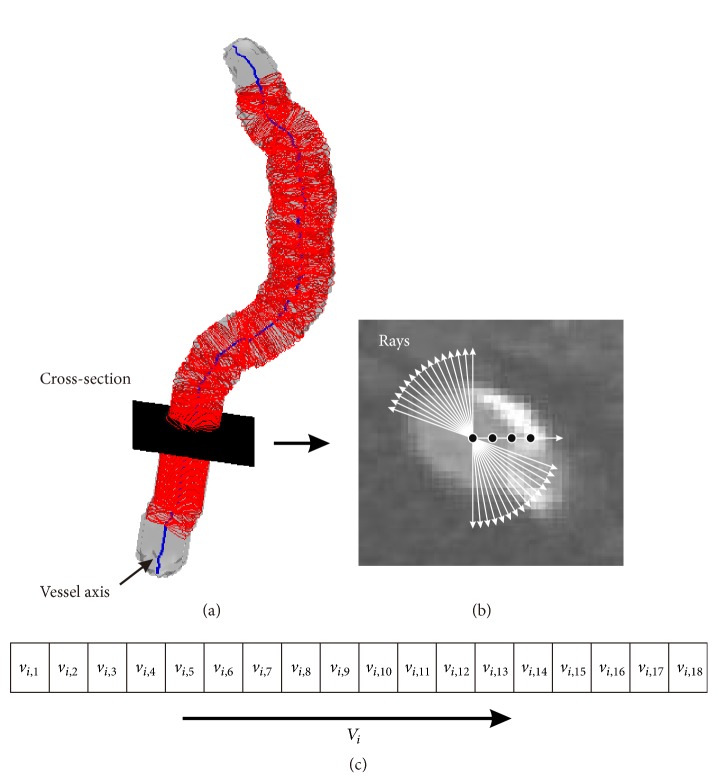
Collecting training samples and testing samples. (a) Vessel cross-section plane orthogonal to the extracted vessel axis. (b) Collecting voxel series along the rays as training samples and testing samples. The black points indicate the voxels on the ray. The rays space every 5° from the axis point. (c) One voxel series along the ray. Along each ray, we collect 18 voxels.

We collect voxel series as training samples and testing samples, based on the segmentations provided by the expert raters. Firstly, vessel axis is extracted by using our previous method [9], see Figure 1(a) for a snapshot. Then, rays are spaced every 5° from the axis point on the cross-section, shown in Figure 1(b). Finally, voxel series are collected along the rays (Figure 1(c)). Along the vessel axes, the voxel series are gathered cross-section by cross-section, to form the training samples and the testing samples. Let us denote all the voxel series as *V* = {*V*_1_,…, *V*_*i*_,…, *V*_*N*_}, where *V*_*i*_ = {*v*_*i*,1_,…, *v*_*i*,*j*_,…, *v*_*i*,*n*_} is a voxel series for one ray, *v*_*i*,*j*_ is a voxel in the voxel series *V*_*i*_, and *N* is the number of voxel series. Please notice that one voxel series is considered as a whole by the proposed MHMM, to describe the tissue transition from the inner vessel to the outside of vessel.

We target at accurate vessel segmentation with the presence of pathologies. However, the pathology obscures the vessel boundary and makes it difficult to identify vessel from pathology. Thus, we divide the voxels into 4 classes: lumen (vessel), intima (vessel boundary), pathology (pathological tissue), and adventitia (outer tissue). By doing so, we can train the MHMM to focus on obscure vessel boundary. To form the vessel boundary class, we strip the outermost layer away from vessel along the ray. Since the vessel wall thickness changes with vessel scale, we change the size of the outermost layer from 2 voxels to 4 voxels, to cover the vessel scale changes, according to the CT scan resolution in this work.

### 2.2. Feature Set for Multiple Hidden Markov Model

Due to the presence of pathologies, it is challenging to segment vessels accurately. And the variance of vessel intensity makes this even worse. In our previous work [26], we found that no vessel feature is optimal in this challenging case. Thus, we employ multiple vessel-specific features here and try to explore the redundant information included in these features, which can cover the widest possible spectrum of situations. Since the diameter (scale) changes in vessels, all the vessel-specific features we chose are multiscale vessel features. These features are extracted by using Hessian matrix, which uses Gaussian convolution to capture the gradient based shape information. And the scale of vessel, *s*, is represented by the standard deviation of Gaussian convolution. In the following, we introduce the multiscale vessel-specific features employed in this paper.

(1) Sato feature (*f*^1^) [5]: this feature is designed for healthy vessel, with bright tubular structure in a dark environment. For one scale, *s*, the Sato vessel feature *f*^1^ can be described as follows:

(1) f1λ1,λ2,s=λ2·exp⁡−λ122α1λ22λ1≤0,  λ2≠0λ2·exp⁡−λ122α2λ22λ1>0,  λ2≠00λ2=0

where *λ*_1_, *λ*_2_, and *λ*_3_ are the eigenvalues of Hessian matrix, subject to *λ*_1_ > *λ*_2_ > *λ*_3_ [5]. These eigenvalues are calculated by using Gaussian convolution, and the scale *s* is the standard deviation for Gaussian convolution; *α*_1_ and *α*_2_ are two preset parameters, *α*_1_ < *α*_2_. In this work, *α*_1_ and *α*_2_ were fixed to 0.5 and 2, respectively.

(2) Frangi feature *f*^2^ [14]: this feature promotes the enhancement of tubular structure and smoothes other structures out. In the original work, this feature gives promising performance to the vessels with low contrast. For a single scale, *s*, the Frangi vessel feature is given as follows:

(2) $$\begin{array}{c} f^{2}\left(\;\lambda_{1},\lambda_{2},\lambda_{3},s\;\right)=\left\{\begin{array}{cc} 0 & \lambda_{2}>0\;\;or\;\;\lambda_{3}>0\\ \left[1-\exp\left(-\frac{R_{A}^{2}}{2\alpha^{2}}\right)\right]\cdot \exp\left(-\frac{R_{B}^{2}}{2\beta^{2}}\right)\cdot \left[1-\exp\left(-\frac{S^{2}}{2\gamma^{2}}\right)\right] & otherwise \end{array}\right. \end{array}$$

where *λ*_1_, *λ*_2_, and *λ*_3_ are defined in (1). The scale *s* is also the standard deviation for Gaussian convolution; *R*_*A*_ = |*λ*_2_|/|*λ*_3_| (subject to *α*) identifies plate-like structures from tubular structures, while $R_{B}=\left|\lambda_{1}\right|/\sqrt{\left|\lambda_{2}\lambda_{3}\right|}$ (subject to *β*) identifies blob-like structures from tubular structures; $S=\sqrt{\lambda_{1}^{2}+\lambda_{2}^{2}+\lambda_{3}^{2}}$ (subject to *γ*) smoothes background noise out. By following the original research, we set the parameters as follows: *α* = 0.5 and *β* = 0.5. Here *γ* is equal to half of the maximum Frobenius norm of the Hessian over all Frobenius norms computed on the whole image.

(3) Shikata feature *f*^3^ [27]: based on Frangi feature, Shikata feature assumes the cross-section of tubular structure has 2-D Gaussian intensity distribution. This assumption gives Shikata feature the capability of detecting small vessels. The feature *f*^3^ is given as follows:

(3) $$\begin{array}{c} f^{3}\left(\;\vec{x},\lambda_{2},s\;\right)=s^{2}\cdot \frac{\lambda_{2}}{I\left(\vec{x}\right)} \end{array}$$

where *λ*_2_ is the second eigenvalues of Hessian matrix, defined in (1) and $I(\vec{x})$ is the intensity at voxel $\vec{x}$.

(4) Li feature *f*^4^ [28]: this feature enhances the tubular structures by using curvature analysis, which gives Li feature the potentiality to distinguish tubular structures from other structures. This Li feature *f*^4^ can be described as follows:

(4) f4λ1,λ2,λ3,s=λ2λ2−λ3λ1λ1<0,  λ2<00otherwise

(5) Manniesing feature *f*^5^ [29]: focusing on background noise and small vessels, this feature improves the Frangi feature, by using a nonlinear anisotropic diffusion approach. This feature, *f*^5^, can be given as follows:

(5) $$\begin{array}{c} f^{5}\left(\;\lambda_{1},\lambda_{2},\lambda_{3},s\;\right)=\left\{\begin{array}{cc} 0 & \lambda_{2}\geq 0\;\;or\;\;\lambda_{3}\geq 0\\ \left[1-\exp\left(-\frac{R_{A}^{2}}{2\alpha^{2}}\right)\right]\cdot \exp\left(-\frac{R_{B}^{2}}{2\beta^{2}}\right)\cdot \left[1-\exp\left(-\frac{S^{2}}{2\gamma^{2}}\right)\right]\exp\left(-\frac{2c^{2}}{\left|\lambda_{2}\right|\lambda_{3}^{2}}\right) & otherwise \end{array}\right. \end{array}$$

where *R*_*A*_ = |*λ*_2_|/|*λ*_3_| is designed to identify plate-like structures from line-like structures, $R_{B}=\left|\lambda_{1}\right|/\sqrt{\left|\lambda_{2}\lambda_{3}\right|}$ has the capability to discriminate blob-like structures from line-like structures, and $S=\sqrt{\lambda_{1}^{2}+\lambda_{2}^{2}+\lambda_{3}^{2}}$ eliminates background noise. By following the original research, we preset *α* = 0.5, *β* = 0.5 in this study. And *γ* is defined in (2).

By following our previous work [26], we set the vessel scale as *s* = 0.6 × 2^(*w*−1)/2^, *w* = 1,2,…, 6, for each of the five features, to cover both large and small vessels (the diameter of vessel changes from 0.7 mm to 6.0 mm). The response of one vessel feature reaches its maximum, when the scale of the feature matches the size of local tubular structures. The maximum response can be collected as the local vessel feature.

For each voxel in the voxel series, we calculate feature values to form feature series. Let us take Sato feature for example. For the voxel series *V*_*i*_ = {*v*_*i*,1_,…, *v*_*i*,*j*_,…, *v*_*i*,*n*_}, we calculate the Sato feature for each voxel in *V*_*i*_ and then obtain a Sato feature series *F*_*i*_^1^ = {*f*_*i*,1_^1^,…, *f*_*i*,*j*_^1^,…, *f*_*i*,*n*_^1^}. *f*_*i*,*j*_^1^ is the Sato feature value corresponding to voxel *v*_*i*,*j*_. After calculating the Sato feature for all the voxel series {*V*_1_,…, *V*_*N*_}, a subset of Sato feature series can be obtained, *F*^1^ = {*F*_1_^1^,…, *F*_*i*_^1^,…, *F*_*N*_^1^}. By using the five proposed vessel features, we finally obtain five subsets of the training/testing samples: *F* = {*F*^1^,…, *F*^*k*^,…, *F*^5^}, where *F*^*k*^ is the *k*th subset corresponding to the *k*th feature, *k* = 1,…, 5. These five subsets are the inputs of MHMM.

### 2.3. HMM-Based Recognition Model

The proposed MHMM is the combination of multidimensional HMMs. Before presenting the MHMM, we would like to introduce one individual HMM first. This HMM is trained by using one of the five features.

HMM is considered to be the best for modeling variations in observations of similar data series and for finding dissimilarities across different series. One HMM (*ϕ*) can be expressed as a five item array as *ϕ* = (*M*, *N*, *π*, *A*, *B*), where *M* is the number of invisible tissue states. In this paper, we set the number of invisible tissue states as 4 to represent four tissue states: lumen (vessel), intima (vessel boundary), pathology (pathological tissue), and adventitia (outer tissue); *N* is the number of observation values, which are decided by the vessel features. We will describe this observation value later; *π*_*k*_ is the initial state distribution *π* = {*π*_*m*_}, *m* = 1,…, *M*. Since we start detecting lumen and intima from the axis point on the cross-section, the initial state distribution is *π*_1_ = 1, *π*_*j*_ = 0, *j* = 2,…, *M*; *A* is the state transition matrix with size *M* × *M*. And *B* is the emission matrix with size *N* × *M*. These two matrixes are the key parameters of HMM, which we are going to estimate in the training process.

**Figure 2:**
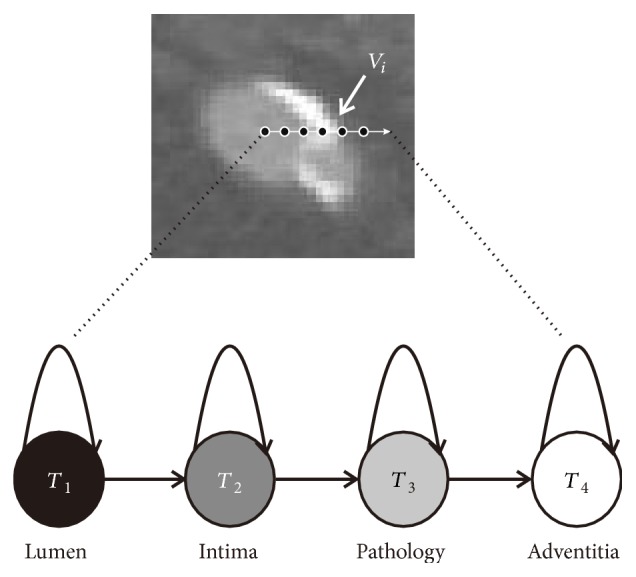
One single HMM, *ϕ*, describes the state transition from inner vessel to the outside of vessel along the ray.

The HMM, *ϕ*, describes the state transition from the inner vessel to the outside of vessel, as shown in Figure 2. This state transition is a visible random process, which gives the similarities between states and voxels. Let us denote the four tissue states (lumen, intima, pathology, and adventitia) as *T* = {*T*_1_,…, *T*_4_}. As shown in Figure 2, we model the pathologies only existing outside the intima. The pathologies we target at are calcification, stenosis, occlusion, and low contrast vessel. Within these pathologies, calcification is a main pathophysiological process in vasculature, and the other pathological tissues come with it. More specifically, calcification determines the location of the other pathological tissues [30]. The location of calcification can be divided into superficial or deep. In the calcification process, deep calcification is followed by plaque shrinkage and arterial remodeling, in which a kind of internal elastic lamina covers the calcification plaque [30]. Based on this finding, many clinical trials consider deep or multiple calcifications as superficial calcification, for blood supply prediction [31]. Thus, we model the pathologies to be outside of the intima.

Different from other algorithms (such as SVM and AdaBoost classifier), the vessel feature values cannot be the input of HMM directly, due to the computational time issue and the convergence issue in the training process [25]. For one vessel feature, the feature values vary in a large area due to different tissues. Associating each feature value with a state will cause a heavy burden for the HMM, which may lead to slow convergence or nonconvergence. Thus, the HMM asks for quantization of feature space into a smaller number of discrete space, called observation space. This quantization is more like a binning process. Firstly, we divide the feature space into several bins. Let us denote the bin set as *θ* = *θ*_1_ ∪ *θ*_2_ … ∪*θ*_*K*_. Then, we associate feature values with bins. If a feature value *a* falls into a bin *θ*_*k*_, *a* ∈ *θ*_*k*_, this feature *a* can be associated with *θ*_*k*_. By associating feature values with bins, we can turn the feature series into a bin series, which is the observation series for HMM.

**Figure 3:**
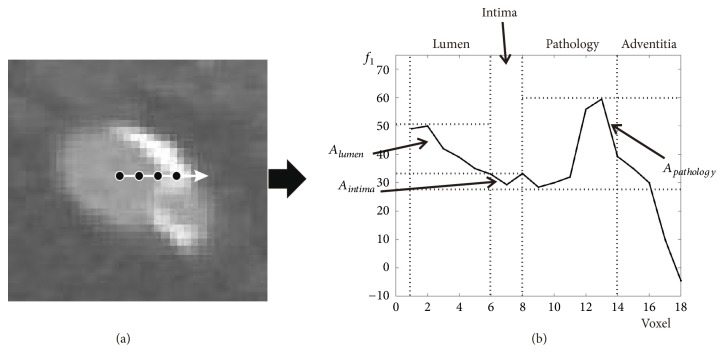
The distribution of Sato feature values in one feature series. (a) The feature series collected along one ray. The black points indicate the voxels we collect along the ray. (b) The distribution of Sato feature values. It can be observed that area *A*_*pathology*_ overlaps *A*_*lumen*_ and *A*_*intima*_.

The method of dividing bins is the key for HMM. In our experiment, we found that the capability of HMM correlates with the number of bins. More specifically, the number of bins determines the capability of HMM in identifying different tissues, in one given area of the feature space. The larger the number of bins is, the more powerful the HMM is. Here, we take the Sato feature, for example, to demonstrate the method we use in dividing bins. Firstly, we look into the distribution of feature values along the ray (Figure 3(a)). Let us denote the distributions of lumen, intima, pathology, and adventitia as *A*_*lumen*_, *A*_*intima*_, *A*_*pathology*_, and *A*_*adventitia*_, respectively. And the distributions of different tissues along the ray are shown in Figure 3(b). It can be easily observed that the distribution of pathology (*A*_*pathology*_) overlaps the distributions of lumen (*A*_*lumen*_) and intima (*A*_*intima*_). Since we target delineating the vessel boundary (intima) accurately with the presence of pathologies, we want the HMM to be powerful in these two areas (*A*_*lumen*_ and *A*_*intima*_). Assume that *A*_*pathology*_ overlaps *A*_*intima*_ and *A*_*lumen*_ in intervals [*a*_1_, *a*_2_) and [*a*_2_, *a*_3_), respectively. We give more bins to the overlapping intervals, to make the HMM more powerful in these areas. The dividing method we use here is shown in Table 1. In this work, we set *K*_1_ and *K*_2_ as *K*_1_ = 5, *K*_2_ = 3 for all the features. The five features we use in this work have different ranges of overlapping areas, which are shown in Table 2. Please notice that the number of bins should not be large, due to the convergence issue. The computation time will be discussed in Section 4.

After turning the feature series into observation series, the HMM, *ϕ* = (*M*, *N*, *π*, *A*, *B*), can be trained by using Baum-Welch algorithm [32]. The initialization condition used for HMM training can be summarized as follows:

1. the number of invisible states *M* = 4;
2. the number of observation values *N* = 10;
3. the initial state distribution *π*_1_ = 1, and *π*_*j*_ = 0, *j* = 2,…, 4;
4. the state transition matrix *A*: uniform distribution by using general principle [25];
5. the emission matrix *B*: uniform distribution by using general principle [25].

### 2.4. Multiple Hidden Markov Model

**Figure 4:**
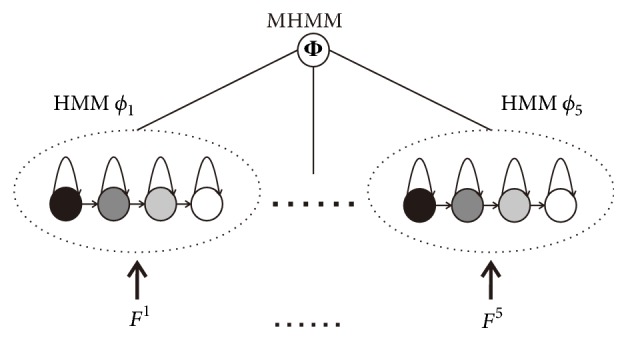
The Multiple Hidden Markov Model (MHMM), Φ, is trained by five subsets {*F*^1^,…, *F*^5^}. These subsets, {*F*^1^,…, *F*^5^}, correspond to the five proposed vessel features {*f*^1^,…, *f*^5^}. Each subset trains a Hidden Markov Model (HMM). And the MHMM is the combination of the five trained HMMs.

Inspired by redundant information (multiple vessel features) [26] and good statistical properties (HMM random process), we formulate the problem of vessel segmentation as the joint segmentation of multidimensional HMMs. Since we target accurate vessel segmentation with the presence of pathologies, covering the widest possible spectrum of challenging situations is the key. We try to combine multidimensional HMMs by using multiple vessel features, as shown in Figure 4. The combination of multidimensional HMMs is the Multiple Hidden Markov Model (MHMM), Φ, which can be given as follows:

(6) $$\begin{array}{c} \Phi =\overset{5}{\underset{k=1}{\sum }}\alpha_{k}\phi_{k} \end{array}$$

where *ϕ*_*k*_ is one HMM trained by using the training subset *F*^*k*^, *k* = 1,…, 5; *α*_*k*_, *k* = 1,…, 5, are nonnegative terms, which give the relative strengths of the respective HMMs. These nonnegative terms are calculated in the training process, which we will describe later.

In the detecting (testing) process, after the testing voxel series *V*′ = {*V*_1_′,…, *V*_72_′} (the rays space every 5°) are collected on the cross-section, feature series *F*′ = {*F*^1′^,…, *F*^5′^} can be calculated for the voxel series, by using the five proposed vessel features. With the feature series *F*′, the trained MHMM Φ can detect vessel boundary (intima) on this cross-section by calculating the posterior probability *P*(*T*∣Φ, *F*′), where *T* = {*T*_1_,…, *T*_4_} are the four tissue states. *P*(*T*∣Φ, *F*′) is a similarity map between the four tissue states and the voxels in the testing voxel series, which is defined as follows:

(7) $$\begin{array}{c} P\left(\;T\;|\;\Phi ,F^{\prime }\;\right)=\overset{5}{\underset{k=1}{\sum }}\alpha_{k}P\left(\;T\;|\;\phi_{k},F^{k^{\prime }}\;\right) \end{array}$$

where *P*(*T*∣*ϕ*_*k*_, *F*^*k*′^) is the similarity map estimated by the *k*th HMM. These similarity maps are calculated by the forward-backward procedures [32], in the same way as for training a HMM. A similarity map for one voxel series, calculated by *P*(*T*∣*ϕ*_1_, *F*^1′^), is shown in Table 3 for example. As the maximums of the similarities are shown in bold, the state transition from the axis point to the outside of vessel can be easily obtained.

For each testing voxel series, we can obtain a state series *T*′ by using maximum likelihood estimation as follows:

(8) $$\begin{array}{c} T^{\prime }=\underset{T=\left\{T_{1},\ldots ,T_{4}\right\}}{\mathrm{argmax}}P\left(\;T\;|\;\Phi ,F^{\prime }\;\right) \end{array}$$

Based on the state series *T*′ (label series), the lumen (vessel) and intima (vessel boundary) can be segmented on the cross-section along the rays. Then along the vessel axes, we can obtain the whole vessel segmentation. Since we collect voxel series along the rays every 5° for training and testing, a given voxel can appear in multiple series and then gets different labels from different series potentially. To handle this multiple labels issue, mean similarity values of the four states are calculated for one given voxel, and the state (label) with maximum value will be signed to this voxel. In our work, we observe a few voxels have equal values in the lumen state and the intima state. Since we consider voxels in these two states as vessel finally, it does not affect the final segmentation result.

As we described in Section 2.3, multidimensional HMMs are trained separately. For feature *f*^*k*^ (training subset *F*^*k*^), the HMM (*ϕ*_*k*_) can be trained by adjusting the parameters of *ϕ*_*k*_ to get the local maximum of the posterior probability *P*(*T*∣*ϕ*_*k*_, *F*^*k*^), by using the Baum-Welch algorithm [32]. Then, the trained HMM (*ϕ*_*k*_) can describe the state transition from the inner vessel to the outside of vessel with the similarity map. And the training precision of this HMM can be estimated by calculating *Precision*_*k*_ = *TP*/(*TP* + *FP*), where *TP* is the true positive representing the number of positive samples detected as positive samples; *FP* is the false positive representing the number of negative samples detected as positive samples. After all the five HMMs have been trained, the nonnegative term *α*_*k*_ for HMM *ϕ*_*k*_ can be defined as *α*_*k*_ = *Precision*_*k*_/∑_*w*=1_^5^*Precision*_*w*_. Finally, their combination (Eq. (6)), the proposed MHMM, can be obtained.

## 3. Results

In order to evaluate the performance of the proposed MHMM, we test it in two challenging cases: (1) synthetic vascular experiment with different levels of Gaussian noise; (2) abdominal artery segmentation with the presence of pathologies. Moreover, we compare our method with two methods to illustrate the capability of our method in delineating accurate vessel boundary with the presence of pathologies.

### 3.1. Two Methods for Comparison

The two chosen methods are state-of-the-art methods: a vessel tractography method proposed by Cetin [33] and a learning-based regression method introduced by Schaap [12]. The vessel tractography method (Cetin's method) is a vessel axis-based method, which combines a vessel tensor with a vessel axis tracing method. This vessel tensor is the key for Cetin's method, which models the vessel as a cylinder. And the learning-based regression method (Schaap's method) can learn the geometry and the appearance of the vessels from annotated data. Notably, these two methods target at geometrical features of vessel. Since the pathologies do not have an explicitly geometrical feature, we believe that these two methods have the capability to identify vessels from pathologies. That is the reason we choose these two state-of-the-art methods for comparison. We implemented these two algorithms ourselves, with changes needed to utilize our clinical datasets, by following the original researches.

Cetin's method and Schaap's method ask a single seed point and an approximate centerline for initializations, respectively. With the vessel axis extracted by using our previous method, we set the start point of the vessel axis as the seed point for Cetin's method and set the vessel axis as the approximate centerline for Schaap's method.

Although Cetin's method is an unsupervised clustering method, some key parameters are estimated by using the training samples as follows: the calcium threshold *t*_*calc*_ = 1500; the height of the cylinder *h* = 4*r*; the number of directions for tensor fitting *m* = 24; the number of directions for branch detection *N* = 256; the initial radius range $\left[\begin{array}{cc} 0.7 & 6 \end{array}\right]$; the termination ratios *β*_0_ = 0.2 and *β*_1_ = 0.9; and the angle parameters *A*_1_ = 5*π*/3 and *A*_2_ = *π*/9.

For the implementation of Schaap's method, several parameters are optimized by following the original optimization sequence [12]: the calcium removal parameters *δ*_*z*_ = 20, *δ*_*i*_ = 300, and *c* = 250; the radius of the region of interest 25 voxels; the expected error of the *K*-nearest neighbors (KNN) estimate *δ*_*knn*_ = 0.25; the number of shape parameters *M* = 12; the number of principal components *P* = 32; and the number of neighbors *K* = 16. The remaining parameters, the regression matrix ${\hat{H}}_{RR}$ and the shape parameters *β*_*x*_^*∗*^, can be trained by using our training samples.

### 3.2. Evaluation Metrics and Statistical Tests

In order to present the comparison results between the proposed MHMM and the two state-of-the-art methods, quantitative analysis is employed to quantify the performance of these three methods, by calculating the value of overlap and accuracy. Here, the manual reference segmentations (Section 2.1) are used as ground truth. Two volume and surface based metrics are borrowed from literatures: Dice Overlap Coefficient (DOC) [34] and Average symmetric Surface Distance (ASD) [35]. The values of DOC and ASD are calculated by comparing the segmented vessels with the manual reference segmentations (ground truth). For DOC (ASD), the larger (smaller) the value is, the better the segmentation result is. And the DOC (ASD) is given in percent (millimeters).

In order to acquire obvious results in comparison, we employ the paired *T*-test, which can assess the differences in segmentation accuracy between the MHMM and the other two methods. The significant differences in comparison are marked with symbol *∗*. And the differences with a level set at *p* < 0.05 will be considered to be statistically significant.

### 3.3. Synthetic Vascular Datasets

**Figure 5:**
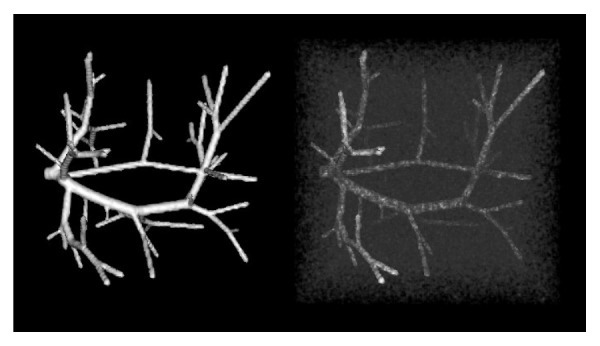
Synthetic vessel tree example before (left) and after (right) adding Gaussian noise.

The synthetic vascular datasets we use here are released by Hamarneh and Jassi [36] in March 2013, which contain 120 datasets. Since the vascular trees in the synthetic vascular datasets are simulated, they can be used as ground truth segmentations. As we discussed previously, the ground truth segmentations of real clinical datasets do not exist, which makes the comparison on synthetic vascular datasets valuable. Furthermore, we simulate the challenging case, the vessel boundary obscured by pathologies, by adding different levels of Gaussian noise.  Figure 5 shows the synthetic vessel tree example before and after adding Gaussian noise.

**Figure 6:**
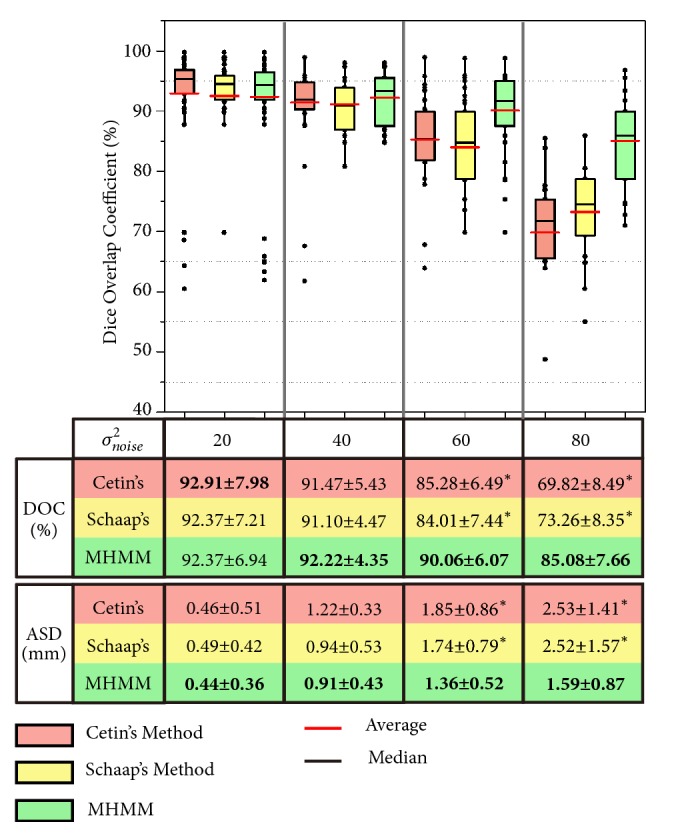
Summary of comparison evaluation on the testing datasets (240 synthetic vascular volumes). Dice overlap coefficients (DOC) using the three methods are plotted. The averages of DOC and average ASD, as well as the statistical significance, are shown in the following table, where the best results are presented in bold, and *∗* indicates the statistically significant differences between our method (MHMM) and the other two methods at a significance level of 0.05.

All the 120 datasets available online are used in our synthetic validation experiments. Gaussian noise with different levels, *σ*_*noise*_^2^ = 20, *σ*_*noise*_^2^ = 40, *σ*_*noise*_^2^ = 60, and *σ*_*noise*_^2^ = 80, is added to the datasets to form the training datasets (240 datasets) and the testing datasets (240 datasets). *σ*_*noise*_^2^ = 20 and *σ*_*noise*_^2^ = 40 are used to simulate the normal noise levels in CT scans [37]. And *σ*_*noise*_^2^ = 60 is for the low dose scans [37]. To our knowledge, noise level changes with body size, and higher noise level can be found in larger body size [38]. Based on this finding, *σ*_*noise*_^2^ = 80 is added to the synthetic datasets, to simulate the real challenging case (low dose scans with large body size). The three methods are tested on the testing datasets. The comparison results are summarized in Figure 6.

Let us focus on the DOC results first. As we can easily observe, the three methods yield similar results, with the presence of low levels of noise (*σ*_*noise*_^2^ = 20 and *σ*_*noise*_^2^ = 40). They all produce a high average DOC, as high as 92.91 ± 7.98%. When the level of noise comes to *σ*_*noise*_^2^ = 60, the average DOC of Cetin's method and Schaap's method goes down dramatically to 85.28 ± 6.49% and 84.01 ± 7.44%, respectively. With the highest level of noise, the situations for these two methods become even worse. However, the performance of our method maintains relatively stable, with the four levels of noise. Our method yields a high average DOC of 92.37 ± 6.94%, 92.22 ± 4.35%, 90.06 ± 6.07%, and 85.08 ± 7.66%.

Then, we present the comparison results by using ASD. An average ASD of 0.46 ± 0.51mm, 1.22 ± 0.33mm, 1.85 ± 0.86mm, and 2.53 ± 1.41mm is obtained by the Cetin's method. These values are changed to 0.49 ± 0.42mm, 0.94 ± 0.53mm, 1.74 ± 0.79mm, and 2.52 ± 1.57mm by the Schaap's method, and the values are improved to 0.44 ± 0.36mm, 0.91 ± 0.43mm, 1.36 ± 0.52mm, and 1.59 ± 0.87mm by our method.

The results summarized in Figure 6 demonstrate that the MHMM can delineate vessel boundary accurately, when the boundary is blurred by high level of noise. Moreover, the MHMM is more resistant to Gaussian noise, compared to Cetin's method and Schaap's method. The differences of DOC and ASD between our method and the other two are statistically significant (*p* < 0.05) with high levels of noise, which are denoted by the symbol *∗*.

**Figure 7:**
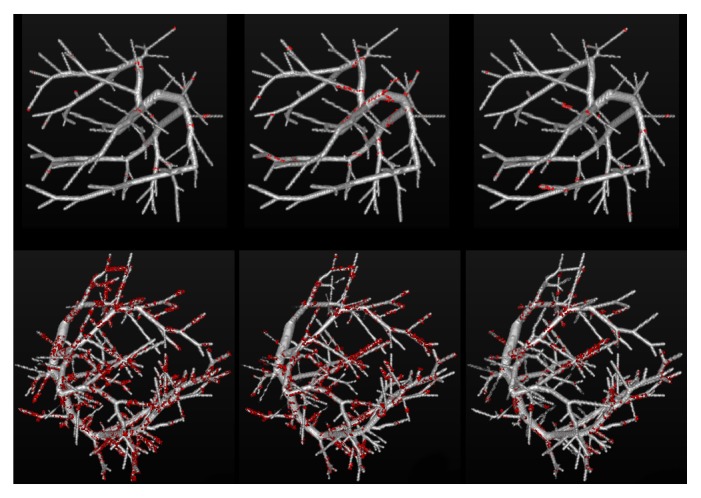
The comparison results on two randomly selected synthetic vascular trees, with the presence of two levels of Gaussian noises *σ*_*noise*_^2^ = 20 (top) and *σ*_*noise*_^2^ = 80 (bottom), respectively. The first column, the second column, and the third column show Cetin's segmentations, Schaap's segmentations, and the segmentations of our method, respectively. The overlapping voxels between one of the three computational methods and the corresponding ground truth are shown in gray, while the voxels detected by the computational methods but do not belong to the ground truth are shown in red.

Figure 7 shows the 3-D views of segmentation results on two randomly selected synthetic vascular trees, with the Gaussian noise of *σ*_*noise*_^2^ = 20 and *σ*_*noise*_^2^ = 80. It can be easily observed that the segmentations obtained by our method are more accurate than those of Cetin's and Schaap's methods.

### 3.4. Abdominal Artery Datasets

**Figure 8:**
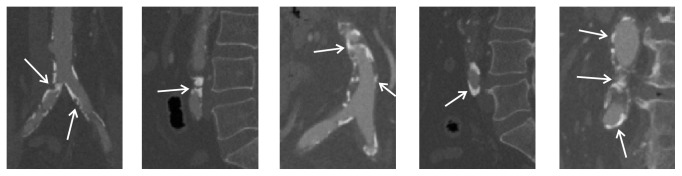
Several challenging cases in abdominal vessel segmentation, caused by extensive vessel calcification. Arrows indicate the vessel calcification, which can result in stenosis, occlusion, low contrast vessel boundary, low contrast vessel, and vessel-like calcification belt.

Recently, there has been a trend towards minimally invasive surgery in the treatment of abdominal artery disease, which means less pain, a shorter hospital stay, and fewer complications. This minimally invasive surgery asks for accurate segmentation of abdominal artery, which can help the surgeon operate with precision, flexibility, and control. However, the extensive vessel calcification in abdominal artery disease obscures the vessel boundary (see Figure 8 for a snapshot) and makes it really challenging to segment vessel accurately. Thus, we collected our abdominal artery datasets from patients with known abdominal artery diseases. We obtained 50 abdominal CT images (120 kV, 40 mAs) by using a Philips Brilliance 64 CT scanner. For all the images, the in-plane resolution varies from 0.65 to 1.00 mm, and the slice thickness varies from 0.5 to 1.0 mm. For training and testing purpose, we randomly divide the 50 abdominal CT images into two datasets. Each of them contains 25 abdominal CT images. Please note that all the 50 abdominal CT images were segmented by two expert raters.

**Figure 9:**
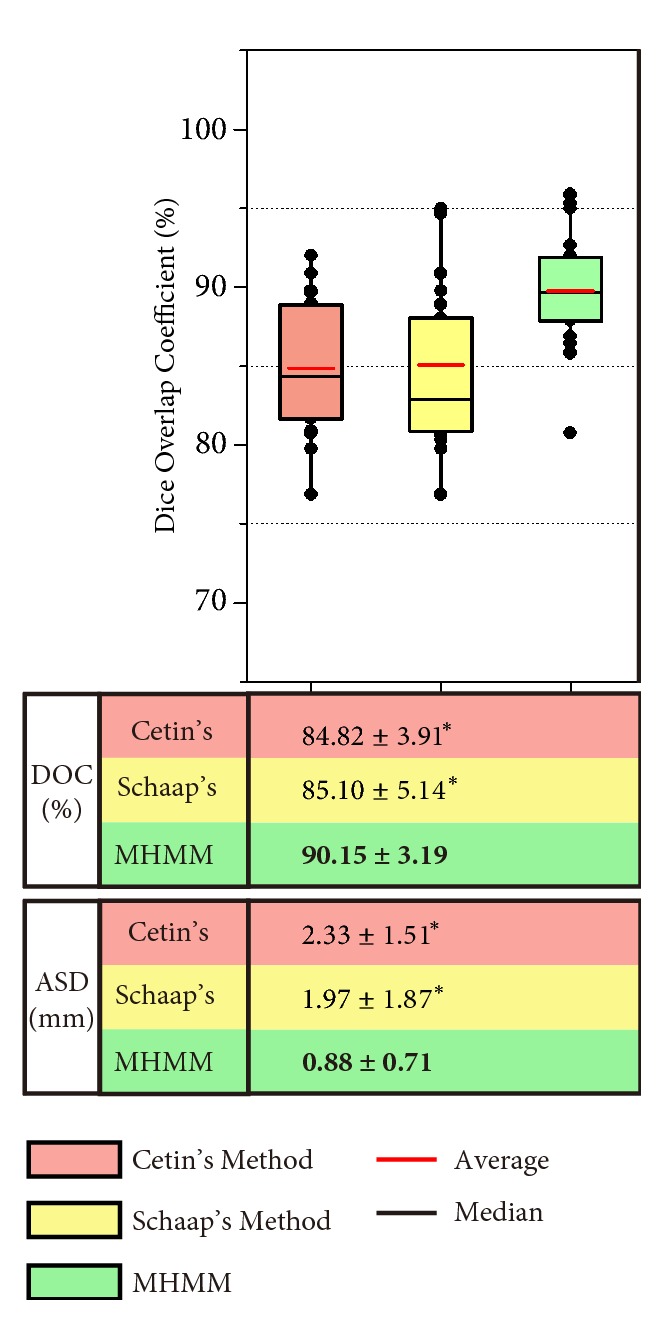
Summary of accuracy evaluation on 30 carotid artery datasets for the three methods. DOCs using Cetin's, Schaap's, and our methods are plotted. Average DOC and ASD values and statistical significance are also shown just below the box plot in a table. In the table, the best results are presented in bold, and symbol *∗* indicates the statistically significant differences between our method and the other two.

The comparison results of the three methods on the abdominal artery datasets are summarized in Figure 9. With the symbol *∗*, we can easily observe that the differences between our method and the other two are statistically significant, in both DOC and ASD. The DOCs are 84.82 ± 3.91%, 85.10 ± 5.14%, and 90.15 ± 3.19% for the Cetin's, Schaap's, and our methods, respectively, while ASDs are 2.33 ± 1.51mm, 1.97 ± 1.87mm, and 0.88 ± 0.71mm. These significant differences indicate our method achieves better segmentation accuracy than the other two state-of-the-art methods.

**Figure 10:**
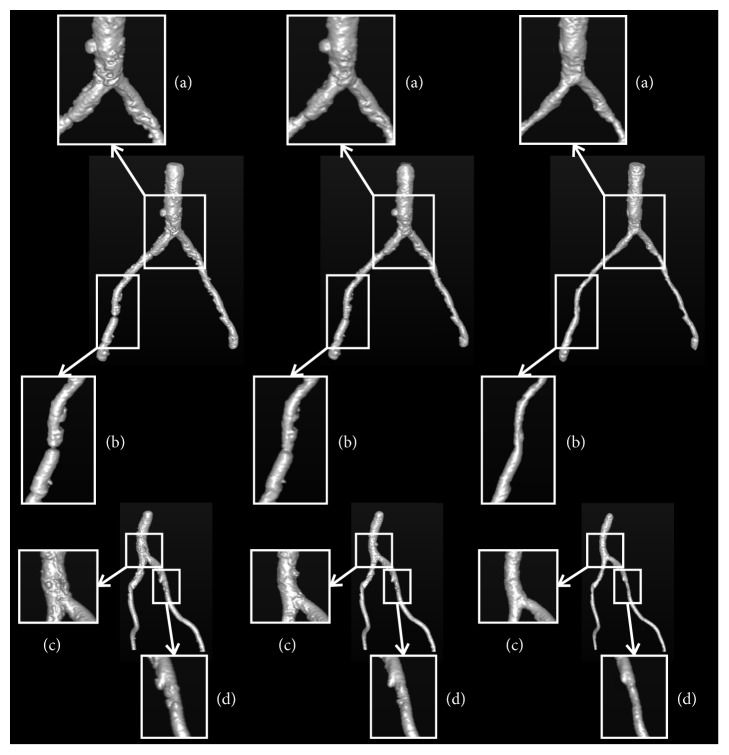
Comparison results of two challenging abdominal artery cases for the three methods. The two rows illustrate the two abdominal arteries. The first column, the second column, and the third column show Cetin's segmentations, Schaap's segmentations, and the segmentations of our method, respectively. The white rectangles indicate the significant differences of the three methods.

**Figure 11:**
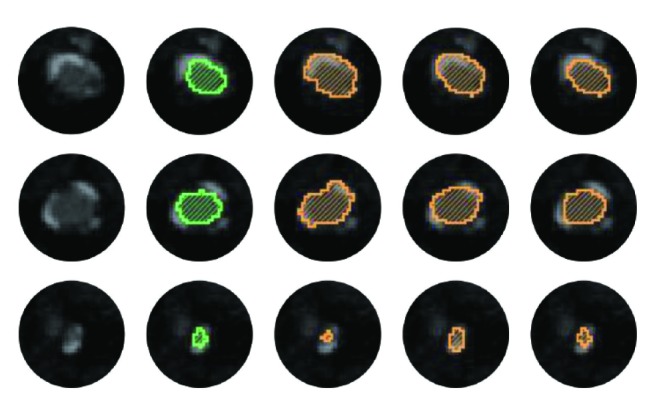
Three examples of 2-D cross-sections with pathologies. The five columns from left to right show the 2-D cross-sections of original images, usersegmented overlays, Cetin's output overlays, Schaap's output overlays, and our output overlays.

As far as we know, the abdominal artery with the presence of pathologies should be segmented accurately for the surgery plan, since the blood supply prediction is critical for the minimally invasive surgery. Here, in order to give an intuitive impression of our method, we present the comparison results in 3-D, Figure 10. Two abdominal arteries shown in Figure 10 are with the challenges in Figure 8. We use the white rectangles to show the comparison areas with significant differences between different segmentations. In comparison area (a) (Figure 10(a)), the vessel kisses a vessel-like calcification belt. Cetin's method and Schaap's method suffer obvious oversegmentation. In the stenosis area (Figure 10(b)), Cetin's method and Schaap's method underestimate or overestimate the radius of vessel. In the low contrast areas (Figures 10(c) and 10(d)), these two methods fail to identify vessel boundary from pathologies. However, our method segments vessel accurately in these challenging cases. These comparison results show that our method has the capability in dealing with the challenging cases caused by pathologies, while the other two methods do not. Moreover, we show several examples of 2-D cross-sections with pathologies in Figure 11. In the calcification areas, the first and second rows in Figure 11, Cetin's and Schaap's methods have more oversegmentations than our method. And in the stenosis area, the third row in Figure 11, Cetin's and Schaap's methods suffer from undersegmentation and oversegmentation, respectively. It can be observed that our method produces the most similar results with the manual reference segmentations, which are considered as ground truth in this paper.

## 4. Discussion

At the very beginning, we tried to model a single HMM with multiple vessel features, by concatenating features to form a large feature vector. However, one single HMM with multiple features suffers from poor performance due to large number of parameters to learn, even nonconvergence [39]. As a result, we failed to train a single HMM with five features by using our datasets. Then, inspired by joint segmentation, we turned to MHMM, which combines multidimensional HMMs.

The proposed MHMM was implemented in Matlab R2016b, with Core i7-8700 (6 cores at 3.2 GHz) and 16 GB RAM, as well as the other two methods. The computation time of the three proposed methods is shown in Table 4 for comparison.

It can be observed that the computation time of our method is much larger than that of the other two methods. Although the proposed MHMM produces accurate segmentations with the presence of pathologies, it suffers from slow convergence. That is the reason we did not apply it to large-scale scans with complex branches, such as lung datasets and brain datasets. In the future, we plan to design a cascade-AdaBoost-MHMM classifier, which organizes the AdaBoost classifiers and MHMM classifiers in a cascade way. This cascade-AdaBoost-MHMM classifier can wipe healthy vessels away, just leaving the problem areas to MHMM.

Although vessel segmentation plays a fundamental role in many clinical fields, the low segmentation accuracy issue caused by pathologies prevents it from widespread use. Several methods [10, 12] have been proposed to deal with some particular challenges, such as low contrast vessel, by using minimal radius based method or area based method. However, these methods fail to identify vessels from pathologies, when facing stenosis and complicated vessel cross-sections. Thus, we propose the MHMM, combining the redundant information (multiple vessel features) and the statistical properties (HMM), to cover the widest possible spectrum of challenging situations.

The absence of a systematic evaluation workflow [40] makes the quantitative validation challenging in this work. It is challenging to compare our method with others, because of different databases, different target populations, and different quality metrics. In order to deal with this challenge and present our method in a more intuitive way, we compare our method with two state-of-the-art algorithms on the same database with the same metrics. We show the comparison results by combining box plots and tables, which gives a comprehensive view of our method. And the comparison results indicate that our method (MHMM) has the capability in segmenting vessels accurately with the presence of pathologies.

## 5. Conclusion

Targeting at vessels with the presence of pathologies, we propose a posterior probability classifier by combining multiple HMMs for accurate vessel segmentation. This classifier is an axis-based method, which works with a vessel axis + cross-section model. This model gives our method two advantages: (1) constraining the classifier around the vessel axes and (2) making the classifier focus on identifying vessels from vessel-like structures. The redundant information that comes with the multiple vessel features helps our method to delineate the obscure vessel boundary accurately. In the synthetic experiment, we use 240 complex-structured datasets (after adding noise) to evaluate the performance of our method. Our method achieves both good DOC and ASD scores, even with the highest level of noise. And the comparison experiment on real clinical datasets makes our method more promising, since the scores achieved by our method are much higher than two state-of-the-art methods.

## Figures and Tables

**Table 1:** Segment method.

Interval	[0, *a*_1_)	[*a*_1_, *a*_2_)	[*a*_2_, *a*_3_)	[*a*_3_, +*∞*)
Dividing method	One bin	Average	Average	One bin

Number of bins	1	*K*_1_	*K*_2_	1

**Table 2:** Overlapping areas for different features.

	[*a*_1_, *a*_2_)	[*a*_2_, *a*_3_)
Sato feature *f*_1_	[25,35)	[35,50)
Frangi feature *f*_2_	[15,20)	[20,30)
Shikata feature *f*_3_	[85,95)	[95,120)
Li feature *f*_4_	[40,45)	[45,55)
Manniesing feature *f*_5_	[15,25)	[25,45)

**Table 3:** A similarity map for one voxel series. The maximums of the similarities are shown in bold.

	*T*_1_	*T*_2_	*T*_3_	*T*_4_	State
*v*_*i*,1_	**0.943**	0.055	0.002	0	*T*_1_
*v*_*i*,2_	**0.901**	0.098	0.001	0	*T*_1_
*v*_*i*,3_	**0.842**	0.153	0.004	0.001	*T*_1_
*v*_*i*,4_	**0.801**	0.195	0.002	0.002	*T*_1_
*v*_*i*,5_	**0.744**	0.254	0.001	0.001	*T*_1_
*v*_*i*,6_	**0.706**	0.290	0.002	0.002	*T*_1_
*v*_*i*,7_	0.301	**0.593**	0.101	0.005	*T*_2_
*v*_*i*,8_	0.001	**0.643**	0.151	0.205	*T*_2_
*v*_*i*,9_	0.007	0.301	**0.572**	0.120	*T*_3_
*v*_*i*,10_	0.001	0.165	**0.732**	0.102	*T*_3_
*v*_*i*,11_	0.002	0.044	**0.812**	0.142	*T*_3_
*v*_*i*,12_	0.001	0.020	**0.509**	0.470	*T*_3_
*v*_*i*,13_	0.001	0.006	0.401	**0.592**	*T*_4_
*v*_*i*,14_	0.002	0.002	0.303	**0.693**	*T*_4_
*v*_*i*,15_	0.001	0.001	0.265	**0.733**	*T*_4_
*v*_*i*,16_	0.001	0.002	0.115	**0.882**	*T*_4_
*v*_*i*,17_	0	0.001	0.109	**0.890**	*T*_4_
*v*_*i*,18_	0	0	0.043	**0.957**	*T*_4_

**Table 4:** Computation time of the three methods.

		Cetin's	Schaap's	Ours
Synthetic	Training	2.2 min	21.2 min	38.6 min
	Testing	6.8 min	24.1 min	32.2 min
Abdominal	Training	1.4 min	11.4 min	25.1 min
	Testing	4.5 min	12.5 min	22.8 min

## Data Availability

(1) The synthetic vascular data used to support the findings of this study are openly available from the Vascusynth Team at http://vascusynth.cs.sfu.ca/Data.html. (2) The crotch artery data used to support the findings of this study have not been made available because the data are owned by a third party (the 2nd Affiliated Hospital of Harbin Medical University).
